# Supplementary material for: Comparative Study of Antiviral, Cytotoxic, Antioxidant Activities, Total Phenolic Profile and Chemical Content of Propolis Samples in Different Colors from Turkiye
Source: Antioxidants (Basel). 2022 Oct 21;11(10):2075. doi: 10.3390/antiox11102075 (PMC9598055; doi:10.3390/antiox11102075)
Supplement: Supplementary file 1 [file antioxidants-11-02075-s001.zip › antioxidants-1975362-supplementary.pdf]

Supplementary Materials

Comparative Study of Antiviral, Cytotoxic, Antioxidant Activities, Total Phenolic Profile and Chemical Content of Propolis Samples in Different Colors from Turkiye

Nazli BOKE SARIKAHYA[1*], Ekin VAROL[2], Gaye SUMER OKKALI[1], Banu YUCEL[2], Rodica MARGAOAN[3*] and Ayse NALBANTSOY[4]

[1]Department of Chemistry, Faculty of Science, Ege University, 35100 Bornova, Izmir, Turkiye
[2]Department of Animal Science, Faculty of Agriculture, Ege University, 35100 Bornova, Izmir, Turkiye.
[3] Advanced Horticultural Research Institute of Transylvania, University of Agricultural Sciences and Veterinary Medicine, 400372 Cluj-Napoca, Romania
[4] Department of Bioengineering, Faculty of Engineering, Ege University, 35100 Bornova, Izmir, Turkiye.
*Corresponding authors: Nazli Boke Sarikahya, Department of Chemistry, Faculty of Science, Ege University, 35100 Bornova, Izmir, Turkiye, nazli.sarikahya@ege.edu.tr
Rodica Margaoan, Advanced Horticultural Research Institute of Transylvania, University of Agricultural Sciences and Veterinary Medicine, 400372 Cluj-Napoca, Romania, rodica.margaoan@usamvcluj.ro

TABLE OF CONTENT

		Page
**Table S1**	The exact collection localities of propolis samples from Turkiye	2
**Table S2**	Compounds and their amounts (mg/g extract) in extracts of propolis from Turkiye	3
**Figure S1**	Cytotoxicity and anti-inflammatory effects of propolis samples	5
**Figure S2**	Physiological changes in the embryos after 48h incubation with propolis-virus mixture	7
Materials and Methods		8

Table S1: The exact collection localities of propolis samples from Turkey and two positive controls propolis samples.

No	The collection areas
**1**	Aydın, Söke
**2**	Aydın, city center
**3**	Manisa, Muradiye
**4**	Sivas, Gürün
**5**	İzmir, Kemalpaşa
**6**	Tekirdağ, city center
**7**	Kars, Digor Varlı (red)
**8**	Kars, city center (green)
**9**	Uşak, city center
**10**	Muğla, city center
**11**	Van, Çatak
**12**	Trabzon, city center
**13**	Manisa, Bozdağ
**14**	Tekirdağ, Çerkezköy
**15**	Burdur, Yeşilova
**16**	Artvin, city center
**17**	Artvin, Aksu
**18**	Erdek, Kapıdağ
**19**	Rize, Çamlıtepe
**20**	İzmir, Bergama/Sarıcalar
**21**	Gümüşhane, Kelkit
**22**	Hakkari, Şemdinli, Korgan
**23**	Kelkit city center, Büyükcami district
**24**	Burdur, Çavdır Vilage
**25**	Iğdır, city center
**26**	Antalya, Serik/Belek
**27**	Tunceli, Ovacık
**28**	Aydın, Çine, Madran
**29**	Iğdır, Tuzluca
**30**	Muğla, Marmaris-İçmeler
**31**	İzmir, Mordoğan
**32**	Bursa, Orhaneli Kuşumlar
**33**	Ankara, city center
**34**	Rize, Kaçkar, Hemşin
**35**	Antalya, Korkuteli
**36**	Bingöl, Kiğı
**37**	Artvin, Borçka-Camili
**38**	İstanbul, Beykoz
**39**	Aydın, Bozdoğan
**40***	Bio-Bee propolis extract
**41***	Brazilian green propolis extract

* Propolis products available on the market as positive control

Table S2: Compounds and their amounts (mg/g extract) in extracts of propolis from Turkey and two positive controls[a-b].

Compounds	Turkh Propolis Extracts																			
	1	2	3	4	5	6	7	8	9	10	11	12	13	14	15	16	17	18	19	20
(-)-Epigallocatechin gallate	<LOD	<LOD	<LOD	<LOD	<LOD	<LOD	<LOD	<LOD	<LOD	<LOD	<LOD	<LOD	<LOD	<LOD	<LOD	<LOD	<LOD	<LOD	<LOD	0.01
(-)-Epigallocatechin	<LOD	<LOD	0.05	<LOD	<LOD	<LOD	<LOD	<LOD	<LOD	<LOD	<LOD	0.01	<LOD	<LOD	<LOD	<LOD	<LOD	<LOD	<LOD	0.05
trans-Taxifolin	0.63	0.05	0.56	0.23	0.26	0.21	<LOD	0.43	0.28	0.63	0.41	0.48	0.29	0.22	0.30	0.11	0.04	0.14	1.17	0.66
3-*O*-Methylquercetin	12.33	3.51	27.91	14.83	14.54	19.04	0.5	22.45	14.89	44.95	12.21	33.39	15.23	8.46	69.44	1.01	2.29	9.45	2.89	94.21
Acacetin	95.96	30.44	49.81	38.53	33.30	52.02	1.25	82.81	33.22	31.87	83.07	83.38	72.60	45.13	165.62	15.31	29.64	34.79	7.25	203.15
Apigenin-7-Glucoside	1.64	<LOD	1.96	0.40	0.58	<LOD	<LOD	0.93	0.60	3.42	0.84	1.91	0.79	0.75	<LOD	<LOD	1.15	<LOD	10.06	0.29
Apigenin	5.36	1.66	5.18	1.39	1.54	5.76	0.63	5.62	1.58	1.50	5.33	9.74	2.88	2.14	2.38	8.44	0.49	0.96	0.76	4.70
Caffeic Acid	9.07	11.15	66.76	42.08	47.11	75.67	0.83	40.54	44.45	14.55	21.58	83.99	19.75	21.09	0.99	12.53	7.14	20.42	0.84	6.01
Chrysin	58.93	49.03	80.27	12.61	12.29	85.66	1.63	69.81	13.80	49.00	52.99	70.40	58.37	56.13	8.62	29.36	21.30	57.85	7.26	30.84
Dihydrokaempferol	<LOD	<LOD	<LOD	5.41	6.31	5.51	<LOD	<LOD	6.18	7.51	<LOD	<LOD	<LOD	<LOD	<LOD	<LOD	<LOD	<LOD	<LOD	<LOD
Ellagic Acid	0.56	1.91	0.80	<LOD	<LOD	0.01	<LOD	<LOD	<LOD	0.08	1.44	0.04	0.34	0.09	0.09	3.36	4.86	7.98	12.87	0.18
Fumaric Acid	0.61	0.52	73.12	0.93	2.12	3.38	<LOD	2.58	1.52	12.82	3.03	47.97	1.10	1.85	1.31	0.84	0.51	1.20	1.14	<LOD
Hederagenin	33.41	163.36	10.69	5.77	18.58	4.85	<LOD	2.92	<LOD	<LOD	78.46	2.76	<LOD	9.57	<LOD	201.92	186.05	<LOD	<LOD	<LOD
Hispidulin	<LOD	<LOD	<LOD	<LOD	<LOD	<LOD	1.65	<LOD	<LOD	<LOD	<LOD	<LOD	<LOD	<LOD	<LOD	<LOD	17.18	<LOD	<LOD	1.48
Hyperoside	0.50	0.34	0.42	0.12	0.12	0.09	5.10	0.17	0.17	1.43	3.87	0.46	0.62	4.41	18.36	0.83	3.91	1.24	22.63	13.64
Isosakuranetin	45.92	359.84	921.32	14.36	11.71	1104.02	92.78	484.24	14.31	329.29	0.00	881.13	352.07	440.83	21.47	133.98	170.78	589.71	45.30	23.95
Myricetin	0.02	<LOD	0.02	<LOD	0.02	0.02	<LOD	<LOD	0.02	0.03	0.03	0.02	0.02	0.02	0.06	3.85	0.38	0.02	0.08	0.09
Naringenin	502.11	188.26	458.17	367.32	304.47	587.51	4.54	721.67	366.79	255.89	494.13	718.66	425.23	338.61	29.10	50.15	107.16	277.43	53.12	134.08
Nepetin-7-glucoside	0.05	0.10	0.24	<LOD	<LOD	0.02	0.31	<LOD	<LOD	9.38	1.79	0.10	0.57	0.39	0.24	<LOD	0.29	0.39	2.46	0.28
Quercetin	8.36	1.37	10.56	4.93	5.96	6.04	<LOD	5.62	5.65	11.48	5.88	17.04	4.34	2.69	13.80	0.88	0.90	2.13	0.47	23.22
Quillaic acid	<LOD	<LOD	<LOD	<LOD	<LOD	<LOD	<LOD	0.03	<LOD	<LOD	<LOD	<LOD	<LOD	<LOD	<LOD	<LOD	<LOD	<LOD	1.46	0.98
Rhamnocitrin	727.06	83.17	274.84	166.19	155.99	70.83	4.72	459.30	188.91	140.86	632.50	403.44	407.57	217.48	301.62	69.79	120.85	135.72	69.33	532.79
Rutin	0.06	0.01	0.36	0.02	0.05	<LOD	<LOD	0.11	0.05	0.47	0.23	0.19	0.04	0.08	1.13	<LOD	0.11	0.37	0.87	0.52
α-Cyano-4-hydroxy cinnamic acid	0.02	0.32	<LOD	<LOD	<LOD	<LOD	<LOD	<LOD	<LOD	<LOD	<LOD	0.06	0.04	<LOD	<LOD	<LOD	<LOD	<LOD	0.01	<LOD
Oleanoic acid	115.61	205.26	59.49	32.78	52.61	46.29	<LOD	84.86	32.75	56.14	327.42	116.79	225.04	52.37	457.90	2478.09	932.96	702.26	92.67	898.27
Chlorogenic Acid	0.03	0.09	0.49	0.02	0.04	0.05	45.21	0.03	0.02	3.74	0.07	0.24	<LOD	<LOD	<LOD	0.25	0.21	<LOD	<LOD	<LOD
Pomolic acid	<LOD	<LOD	<LOD	15.27	12.46	13.91	<LOD	18.48	13.95	8.13	<LOD	<LOD	<LOD	<LOD	16.33	<LOD	<LOD	62.24	<LOD	37.24
Tormentic acid	<LOD	539.78	<LOD	<LOD	<LOD	<LOD	<LOD	6.61	<LOD	22.43	<LOD	<LOD	<LOD	1447.61	56.13	777.84	427.28	66.91	<LOD	<LOD
Diosmetin	12261.63	235.00	4787.1	1900.43	2158.36	2206.93	80.34	6471.67	2331.64	1902.43	7639.07	9106.54	7189.31	3234.07	5799.68	<LOD	599.82	530.35	777.02	9436.89
trans-4-Hydroxy cinnamic acid	3.61	20.67	15.53	15.45	15.43	30.03	<LOD	10.95	10.54	2.02	2.62	32.04	6.74	7.83	<LOD	62.41	29.83	19.30	0.11	0.48
Sinensetin	<LOD	<LOD	<LOD	<LOD	<LOD	<LOD	<LOD	<LOD	<LOD	<LOD	<LOD	0.06	<LOD	<LOD	<LOD	<LOD	<LOD	<LOD	<LOD	<LOD

Compounds	Turkish Propolis Extrats																				
	**21**	**22**	**23**	**24**	**25**	**26**	**27**	**28**	**29**	**30**	**31**	**32**	**33**	**34**	**35**	**36**	**37**	**38**	**39**	**40***	**41***
(-)-Epigallocatechin gallate	0.01	<LOD	<LOD	<LOD	<LOD	<LOD	<LOD	<LOD	<LOD	<LOD	<LOD	<LOD	<LOD	<LOD	<LOD	<LOD	<LOD	<LOD	<LOD	<LOD	<LOD
(-)-Epigallocatechin	0.01	<LOD	<LOD	<LOD	<LOD	<LOD	<LOD	<LOD	<LOD	<LOD	<LOD	<LOD	<LOD	<LOD	<LOD	<LOD	<LOD	<LOD	<LOD	<LOD	<LOD
trans-Taxifolin	0.37	3.06	0.38	0.14	0.37	0.24	0.22	0.30	0.62	0.04	<LOD	0.41	0.23	0.66	0.09	0.16	0.02	0.23	0.45	0.09	0.04
3-*O*-Methylquercetin	17.48	106.69	16.68	20.43	21.76	27.51	10.16	2.03	107.83	1.76	31.32	30.88	24.80	2.83	3.21	20.20	2.23	13.74	28.57	5.38	3.17
Acacetin	37.98	1189.69	61.24	57.43	100.28	55.32	39.82	19.70	94.06	11.92	112.10	39.82	69.75	7.31	1.72	47.68	9.04	54.77	55.98	39.04	0.49
Apigenin-7-Glucoside	<LOD	10.93	<LOD	0.17	0.53	0.47	<LOD	0.14	<LOD	0.08	2.56	7.27	4.39	0.52	0.39	<LOD	<LOD	<LOD	<LOD	0.27	0.17
Apigenin	4.38	67.35	5.94	1.51	7.71	3.32	2.93	0.69	4.44	0.14	5.57	3.12	2.68	0.37	<LOD	3.43	0.40	3.54	4.84	1.78	0.21
Caffeic Acid	58.99	636.09	52.58	10.18	34.02	63.73	26.38	6.04	7.74	0.98	0.90	127.25	63.77	0.97	0.66	26.82	4.98	37.34	34.24	17.57	8.57
Chrysin	32.71	685.76	62.05	42.74	71.79	122.95	36.95	26.28	83.66	22.79	63.65	80.37	99.42	8.86	3.18	27.91	7.48	69.40	65.06	27.77	0.16
Dihydrokaempferol	<LOD	<LOD	<LOD	<LOD	<LOD	<LOD	<LOD	<LOD	<LOD	<LOD	<LOD	<LOD	<LOD	<LOD	<LOD	<LOD	<LOD	10.08	<LOD	<LOD	3.21
Ellagic Acid	0.02	0.28	0.17	0.09	<LOD	0.01	0.73	0.12	0.64	0.21	0.01	1.01	0.01	2.01	<LOD	0.72	21.69	9.48	2.40	<LOD	0.32
Fumaric Acid	33.14	57.03	0.82	1.39	0.93	24.67	1.49	<LOD	<LOD	7.16	<LOD	17.45	48.23	0.73	<LOD	0.71	8.05	1.23	0.29	0.91	0.98
Hederagenin	5.17	123.12	16.91	25.35	10.10	3.32	566.58	24.07	109.97	142.34	<LOD	<LOD	3.93	<LOD	7.54	621.16	<LOD	<LOD	<LOD	1.57	<LOD
Hispidulin	<LOD	<LOD	0.19	<LOD	<LOD	<LOD	<LOD	<LOD	0.51	0.01	<LOD	<LOD	<LOD	<LOD	<LOD	<LOD	33.70	<LOD	<LOD	<LOD	<LOD
Hyperoside	0.20	1.74	0.18	1.79	0.13	0.16	4.47	0.20	1.23	2.83	0.13	0.61	0.25	13.41	0.12	1.37	7.36	0.41	3.01	0.06	2.48
Isosakuranetin	308.91	516.96	599.11	196.66	78.95	1360.42	15.83	23.11	51.76	53.82	645.92	1241.66	1358.47	99.56	5.42	15.92	12.40	842.63	340.64	80.37	4.41
Myricetin	0.01	0.30	<LOD	0.04	<LOD	0.03	<LOD	<LOD	0.69	0.02	0.14	0.01	0.02	0.01	0.02	0.05	0.03	0.06	0.02	0.01	<LOD
Naringenin	370.99	6384.25	576.38	237.85	536.91	660.63	330.20	108.69	1349.52	89.83	422.42	547.19	735.96	67.47	6.28	384.05	19.61	482.37	415.79	230.63	10.55
Nepetin-7-glucoside	0.04	0.31	0.07	0.08	<LOD	0.01	7.51	<LOD	0.64	0.36	<LOD	0.13	0.06	2.07	0.41	0.81	0.70	0.21	0.19	0.02	<LOD
Quercetin	4.86	54.52	6.35	4.05	3.96	4.25	1.72	0.42	17.43	0.70	5.56	12.74	5.16	0.44	1.72	3.47	1.39	6.81	11.51	1.21	0.63
Quillaic acid	0.04	0.23	0.45	<LOD	<LOD	<LOD	<LOD	<LOD	<LOD	<LOD	<LOD	<LOD	0.07	0.48	<LOD	8.45	<LOD	1.48	0.11	<LOD	<LOD
Rhamnocitrin	199.56	6117.52	413.89	236.24	451.28	189.25	258.16	131.37	1426.19	201.00	327.90	96.07	194.76	66.49	9.50	275.22	57.48	262.45	446.91	210.43	580.96
Rutin	0.10	<LOD	0.04	0.08	0.02	0.01	0.14	0.01	0.40	0.02	<LOD	0.03	0.02	0.56	<LOD	0.30	0.38	0.20	0.10	0.01	0.25
Verbascoside	<LOD	<LOD	<LOD	<LOD	<LOD	<LOD	<LOD	<LOD	<LOD	<LOD	<LOD	<LOD	<LOD	<LOD	<LOD	0.01	<LOD	<LOD	<LOD	<LOD	<LOD
Oleanoic acid	166.32	843.50	135.08	192.73	78.35	55.77	894.62	18.38	956.54	220.65	<LOD	<LOD	<LOD	<LOD	<LOD	472.07	<LOD	266.78	245.21	31.76	15.12
Chlorogenic Acid	<LOD	<LOD	<LOD	<LOD	0.01	0.47	0.03	<LOD	0.16	0.70	<LOD	0.05	0.02	0.13	0.09	0.16	0.22	0.09	0.03	<LOD	11.23
Pomolic acid	19.01	<LOD	<LOD	<LOD	14.61	19.11	<LOD	32.37	625.42	289.49	7.31	41.74	15.63	26.19	12.21	<LOD	<LOD	<LOD	<LOD	<LOD	<LOD
Tormentic acid	<LOD	109.86	123.11	121.84	1.08	<LOD	3016.61	<LOD	490.90	394.74	<LOD	<LOD	<LOD	<LOD	<LOD	<LOD	<LOD	4222.48	<LOD	42.44	<LOD
Diosmetin	5398.77	103392.8	6992.39	3879.98	6702.54	2251.29	4124.90	3027.05	28448.7	4238.25	7910.37	3190.04	6622.21	1325.52	21.02	9230.18	1332.22	7269.36	15954.74	2950.04	7930.49
trans-4-Hydroxycinnamic acid	21.20	77.44	17.14	3.68	12.94	11.40	5.22	1.79	<LOD	1.76	<LOD	63.05	19.65	1.62	<LOD	6.94	31.55	34.33	10.01	4.11	36.61
Sinensetin	<LOD	<LOD	<LOD	<LOD	<LOD	0.06	<LOD	<LOD	<LOD	<LOD	<LOD	0.04	<LOD	<LOD	<LOD	<LOD	<LOD	<LOD	<LOD	<LOD	<LOD

[a]standard commercial propolis samples

[b] Data are taken from literature [32].

A

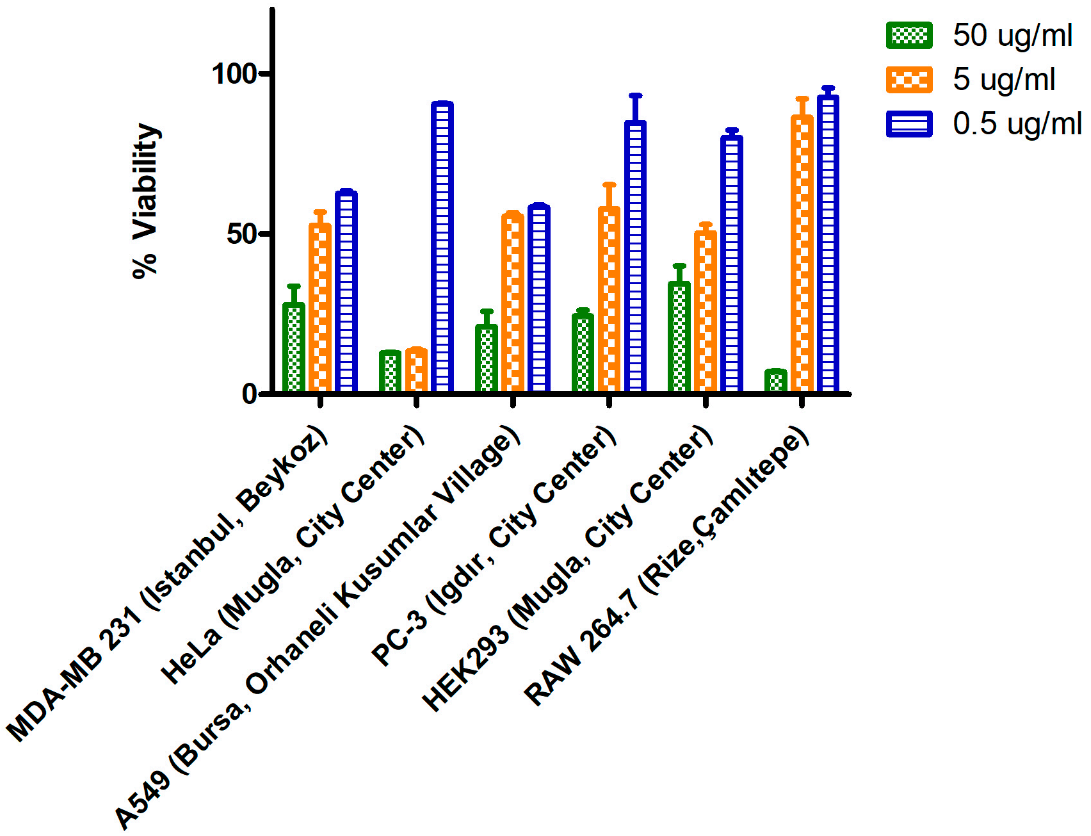

% Viability
100
50
0
50 ug/ml
5 ug/ml
0.5 ug/ml
MDA-MB 231 (Istanbul, Beykoz)
HeLa (Mugla, City Center)
A549 (Bursa, Orhaneli Kusumlar Village)
PC-3 (Igdır, City Center)
HEK293 (Mugla, City Center)
RAW 264.7 (Rize,Çamlıtepe)

B

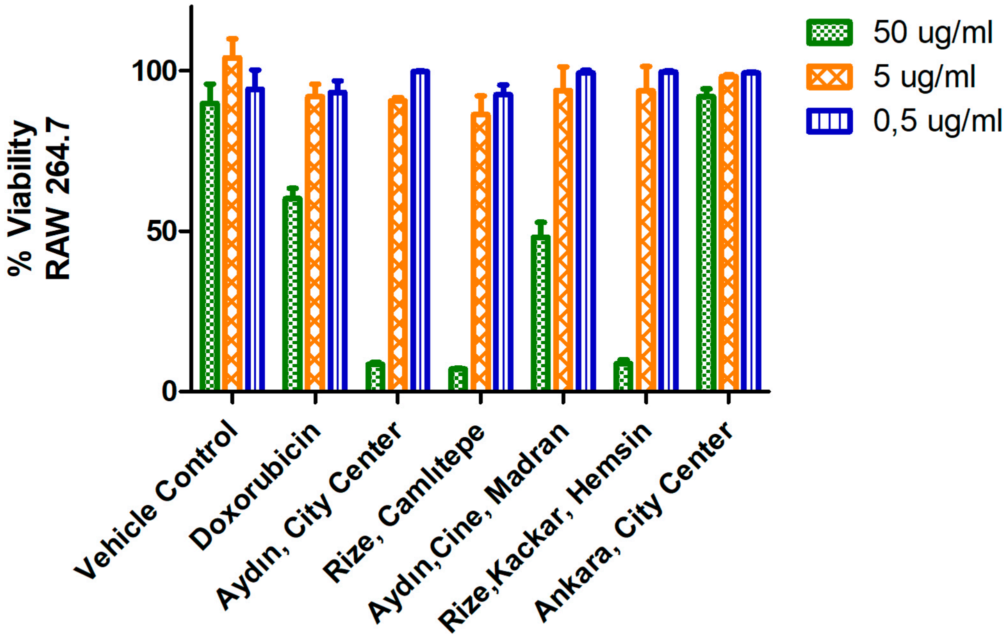

% Viability
RAW 264.7
100
50
0
50 ug/ml
5 ug/ml
0,5 ug/ml
Vehicle Control
Doxorubicin
Aydın, City Center
Rize, Camlıtepe
Aydın,Cine, Madran
Rize,Kackar, Hemsin
Ankara, City Center

C

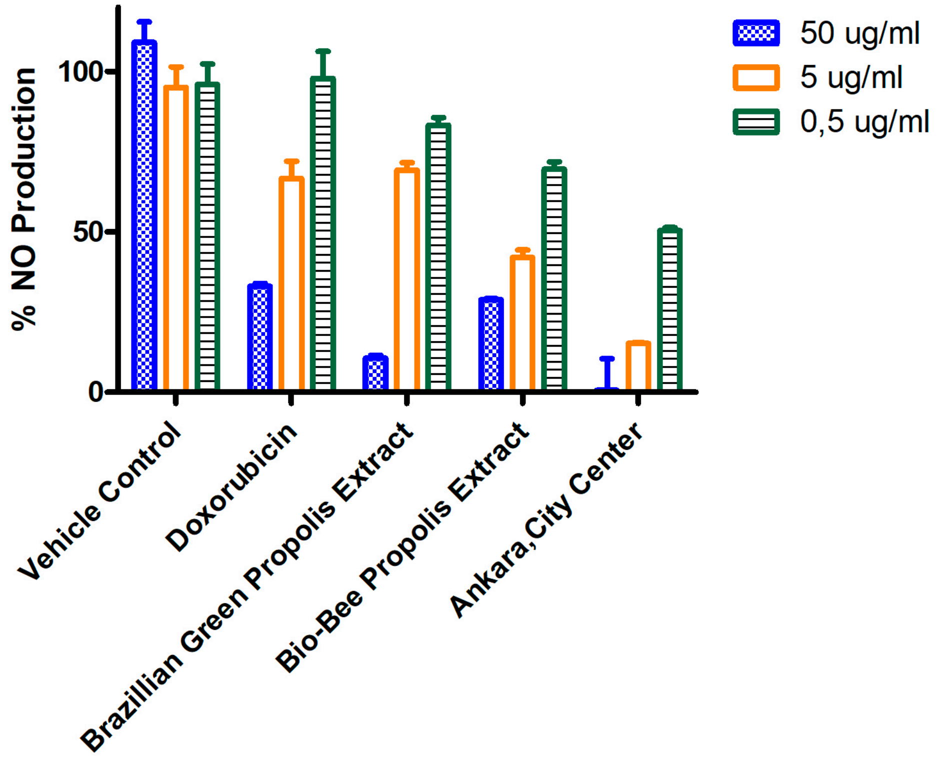

Figure S1: Cytotoxicity and anti-inflammatory effects of propolis samples A) % Based on highest cytotoxicity results of propolis samples, cell viability graph on cancer, non-tumoral and macrophage cells B) Propolis samples that reached IC_{50} value as a result of MTT test in macrophage cells and inhibited NO production C) % Nitric Oxide (NO) production on LPS-activated RAW 264.7 macrophage cells positive controls doxorubicin, Brazillian green propolis extract, Bio-Bee Propolis Extract and the most effective propolis sample 33 Ankara, City Center. Doxorubicin 20, 2, and 0.2 µg/ml was used. Control was exposed to vehicle only which was taken as 100 % viability. Data are expressed as mean ± SD [32].

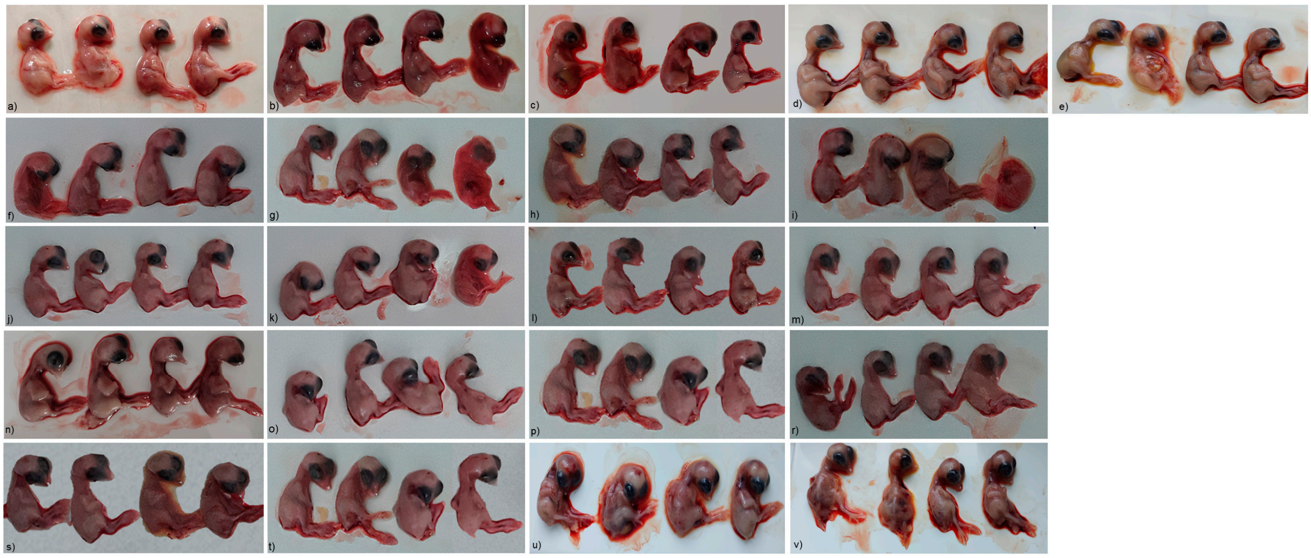

Figure S2: Physiological changes in the embryos after 48h incubation with propolis-virus mixture a) Negative untreated SPF-ECE control b) Negative control, only virus c) Negative vehicle control, treated with %5 DMSO d) Positive control, antiviral agent favipiravir, 10 µg/g e) Positive control, antiviral agent favipiravir, favipiravir 25 µg/g f) Propolis Uşak sample, 0.1 µg/g, g) Propolis Uşak sample, 1 µg/g, h) Propolis Çatak-Van sample, 0.1 µg/g, i) Propolis Çatak-Van sample, 1 µg/g, j) Propolis Çerkezköy-Tekirdağ sample, 0.1 µg/g, k) Propolis Çerkezköy-Tekirdağ sample, 1 µg/g, l) Propolis Çamlıtepe-Rize sample, 0.1 µg/g, m) Propolis Çamlıtepe-Rize sample, 1 µg/g, n) Propolis Şemdinli-Hakkari sample, 0.1 µg/g, o) Propolis Şemdinli-Hakkari sample, 1 µg/g, p) Propolis Serik-Antalya sample, 0.1 µg/g, r) Propolis Serik-Antalya sample, 1 µg/g, s) Propolis İçmeler-Marmaris sample, 0.1 µg/g, t) Propolis İçmeler-Marmaris sample, 1 µg/g, u) Propolis Borcka-Artvin sample, 0.1 µg/g, v) Propolis Borcka-Artvin sample, 1 µg/g [32].

Materials and Methods

Preparation of samples for LC-HRMS Analysis

The dried 50-100 mg of the ethanol-water extracts of propolis were dissolved in water in a 5 ml volumetric flask. The flask was kept in an ultrasonic bath until a clear solution was obtained. Then, 100 µL of dihydrocapsaicin solution (from 100 ppm stock solution) was added as an internal standard and diluted to the volume with mobile phase and mixed and warmly heated to get clear solution. The solution was filtered through a 0.45 µm Millipore Millex-HV filter and the concentration of final solution (1 mL) was transferred into a capped auto sampler vial, from which 2 µL of sample was injected to LC for each run. The samples in the auto sampler were kept at 15ºC during the experiment [32].

Instruments and Chromatographic Conditions of LC-HRMS

LC-HRMS experiments were achieved on a Thermo ORBITRAP Q-EXACTIVE mass spectrometry equipped with a Troyasil C18 column (150 x 3 mm i.d., 3 µm particle size). The mobile phases A and B were composed of 1% formic acid-water and 1% formic acid-methanol, respectively. The gradient programme of which was 0-1.00 min 50% A and 50% B, 1.01-6.00 min 100% B, and finally 6.01-10 min 50% A and 50% B. The flow rate of the mobile phase was 0.35 mL/min, and the column temperature was set to 22°C. Environmental conditions were set as temperature 22.0 ± 5.0 °C and relative humidity (50 ± 15) % rh [32].

Optimization of HPLC Methods and LC-HRMS Procedure

The validation parameters consisted of linearity, repeatability, recovery, limit of detection (LOD) and limit of quantification (LOQ) experiments. The linearity for each compound was determined by analysing standard solution. Repeatability, and intermediate precision of the applied method used and screening measurements were carried out by using the extract of sample of Tekirdag propolis and the data was considered during the uncertainty budget estimation of the method as described in previous report. Recovery data of components were evaluated according to the detected levels of compounds in the Tekirdag propolis, we spiked the extract to reach the final concentrations as 0.1 mg/L and 0.5 mg/L and 1 mg/L and the extracts were dissolved in 5 mL volumetric flasks. Unspiked plant extracts were also analyzed to determine the target compounds concentrations in the blank sample. The recovery data of each individual compounds at each fortification levels was calculated according to the following formula [32].

$$Recovery\ \% = \frac{Measured\ concentration - endogeneous\ concentration}{spiked\ concentration} x100$$

The best mobile phase was determined to be an acidified methanol and water gradient in HPLC method. This mobile phase was also found to be suitable for ionization abundance and separation of compounds. The best ionization of small and relatively polar compounds was obtained by ESI source. The ions between m/z 85-1500 were scanned in high resolution mode of instrument. Identification of compounds (Table S3) was done by comparison of retention time of standard compounds (in the range of purity 95% - 99% see section chemicals) and HRMS data of Bezmialem Vakif University, Drug Application and Research Center Library (ILMER). Dihydrocapsaicin (purity 95 %) used as an internal standard for LC-HRMS measurements in order to reduce to repeatability problem of caused by external effects, such as ionization repeatablity, in mass spectrometry measurements. The detailed mass parameter of each target compound was given in Table S2 [32].
